# Inhibition of iduronic acid biosynthesis by ebselen reduces glycosaminoglycan accumulation in mucopolysaccharidosis type I fibroblasts

**DOI:** 10.1093/glycob/cwab066

**Published:** 2021-06-29

**Authors:** Marco Maccarana, Emil Tykesson, Edgar M Pera, Nadège Gouignard, Jianping Fang, Anders Malmström, Giancarlo Ghiselli, Jin-ping Li

**Affiliations:** Department of Medical Biochemistry and Microbiology, BMC B11, Uppsala University, Husargatan 3 Box 582 751 23 Uppsala, Sweden; Department of Experimental Medical Science, BMC C12, Lund University, BMC H11, 221 84 Lund, Sweden; Department of Experimental Medical Science, BMC C12, Lund University, BMC H11, 221 84 Lund, Sweden; Department of Laboratory Medicine, Lund Stem Cell Center, Lund University, BMC H11, 221 84 Lund, Sweden; Department of Laboratory Medicine, Lund Stem Cell Center, Lund University, BMC H11, 221 84 Lund, Sweden; GlycoNovo Technologies Co., Ltd., Shanghai 201203, China; Department of Experimental Medical Science, BMC C12, Lund University, BMC H11, 221 84 Lund, Sweden; Glyconova Srl, Parco Scientifico Silvano Fumero, Bioindustry Park Silvano Fumero S.p.A Via Ribes, 5 - 10010 - Colleretto Giacosa (TO), Italy; Department of Medical Biochemistry and Microbiology, BMC B11, Uppsala University, Husargatan 3 Box 582 751 23 Uppsala, Sweden

**Keywords:** chondroitin dermatan sulfate, ebselen, epimerases, mucopolysaccharidosis type I, substrate reduction therapy

## Abstract

Mucopolysaccharidosis type I (MPS-I) is a rare lysosomal storage disorder caused by deficiency of the enzyme alpha-L-iduronidase, which removes iduronic acid in both chondroitin/dermatan sulfate (CS/DS) and heparan sulfate (HS) and thereby contributes to the catabolism of glycosaminoglycans (GAGs). To ameliorate this genetic defect, the patients are currently treated by enzyme replacement and bone marrow transplantation, which have a number of drawbacks. This study was designed to develop an alternative treatment by inhibition of iduronic acid formation. By screening the Prestwick drug library, we identified ebselen as a potent inhibitor of enzymes that produce iduronic acid in CS/DS and HS. Ebselen efficiently inhibited iduronic acid formation during CS/DS synthesis in cultured fibroblasts. Treatment of MPS-I fibroblasts with ebselen not only reduced accumulation of CS/DS but also promoted GAG degradation. In early *Xenopus* embryos, this drug phenocopied the effect of downregulation of DS-epimerase 1, the main enzyme responsible for iduronic production in CS/DS, suggesting that ebselen inhibits iduronic acid production in vivo. However, ebselen failed to ameliorate the CS/DS and GAG burden in MPS-I mice. Nevertheless, the results propose a potential of iduronic acid substrate reduction therapy for MPS-I patients.

## Introduction

Mucopolysaccharidosis type I (MPS-I) is a progressively debilitating disorder which is caused by mutations of the gene encoding alpha-L-iduronidase (IDUA), an enzyme important for the degradation of glycosaminoglycans (GAGs) in the lysosomes (Hampe et al. 2020). Without the proper amount of this enzyme, GAGs, including chondroitin/dermatan sulfate (CS/DS) and heparan sulfate (HS), accumulate in lysosomes and in the extracellular matrix (ECM), leading to impaired cellular functions in many organs. Although MPS-I is a rare disease, the genetic defect affects children, causing a huge family and social burden. Improved clinical status and prognosis of MPS-I patients are brought by the two available options: hematopoietic stem cell transplantation and replacement of the enzyme. However, these treatments have several disadvantages, including limited effects in some organs, e.g. the brain and cardiovascular system (Chen et al. 2019). Moreover, the active enzyme needs to be infused into the patient, with the potential risk of immune responses. Due to the high cost of enzyme production and transplantation procedure, these strategies have been exclusively limited to wealthy countries. Therefore, the development of cheaper and easy-to-use medicines is desirable for all MPS patients.

GAGs are synthesized in the Golgi apparatus by an array of enzymes, including glucuronyl C5-epimerases (DS-epi1 and DS-epi2 for CS/DS and Hsepi for HS), which convert glucuronic acid to iduronic acid, and several sulfotransferases which stabilize iduronic acid by subsequent sulfation at C2 in HS, or by 4-O-sulfation of adjacent *N*-acetyl-galactosamine residues in CS/DS (Prechoux et al. 2015; Tykesson et al. 2018). The GAGs are then transported to the cell surface and ECM from where they can be internalized and degraded by enzymes, including IDUA in lysosomes.

Not only an excess but also too low amounts of GAGs can cause medical problems. This is illustrated by the musculocontractural type of Ehlers-Danlos syndrome (MCEDS) which exhibits connective tissue fragility, congenital malformations and distinct craniofacial features (Kosho 2016). This rare disorder is caused by mutations in genes that encode CS/DS-biosynthetic enzymes, including dermatan-4-*O*-sulfotransferase 1 (D4ST1) or DS-epi1, encoded by the gene DSE. Dse-knockout mice have connective tissue fragility due to CS/DS alteration, which impairs the assembly of collagen fibrils (Maccarana et al. 2009). In *Xenopus* embryos, DS-epi1 has an important function in the migration of neural crest (NC) cells (Gouignard et al. 2016). The NC is a highly motile stem cell population in vertebrate embryos, which give rise to a variety of cell types, such as peripheral neurons, melanocytes or cartilage and bone in the head (Mayor and Theveneau 2013). The findings that CS/DS is essential for *Xenopus* NC cells to adhere to fibronectin and to migrate in the embryo suggest that a defect in NC cell development might contribute to the craniofacial phenotype in MCEDS (Gouignard et al. 2016).

Ebselen (2-phenyl-1,2-benzoisoselenazol-3(2H)-one) is a synthetic drug which is reported to target multiple biological pathways, including anti-oxidation and anti-inflammation activities (Azad and Tomar 2014). A recent study proposed ebselen as a lead compound for treatment of COVID-19 based on its activity in inhibition of the main SARS-CoV-2 protease M^pro^ (Sies and Parnham 2020). In this study, through screening the Prestwick drug library, we have identified ebselen as a potent inhibitor of glucuronyl C5-epimerases involved in the biosynthesis of CS/DS and HS. Using *Xenopus* embryos as a model, we show that ebselen mimics the effect of Dse knockdown and inhibits the migration of NC cells. Application of ebselen significantly reduced accumulation of the CS/DS in the lysosomes of MPS-I patient-derived fibroblasts. However, the treatment with the compound failed to reduce CS/DS and GAG accumulation in an MPS-I mouse model.

## Results

### Ebselen inhibits the activity of enzymes involved in iduronic acid biosynthesis

**Fig. 1:**
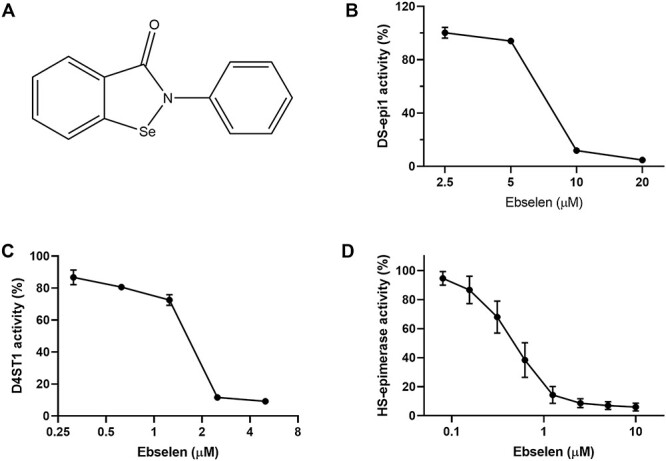
Ebselen inhibits the activities of DS epimerase 1 (DS-epi1), DS 4-*O*-sulfotransferase 1 (D4ST1) and heparan sulfate epimerase (HSepi). (**A**) Chemical structure of ebselen. (**B**–**D**) Activity assays in the presence of ebselen. Data (*n* = 3) are expressed as percentage of remaining activity compared to control. The calculated IC_50_ was 7 μM for DS-epi1, 1.5 μM for D4ST1 and 0.8 μM for HSepi.

The initial aim of this study was to identify compounds that inhibit DS-epi1, which is the major enzyme in the biosynthesis of iduronic acid in CS/DS. The screening of the Prestwick library, containing 1200 drugs or prospective drugs, identified 15 compounds which inhibited DS-epi1 activity by at least 30% at a concentration of 50 μM. Among these, ebselen (Figure 1A) and amphotericin B were most efficient and showed more than 90% inhibition of DS-epi1 activity.

Since ebselen has well-studied and favorable pharmacokinetic profile (Azad and Tomar 2014), we selected this compound for further studies. Apart from DS-epi1 used in the first screening, we found that ebselen also displayed inhibitory activity on D4ST1 (sulfotransferase in CS/DS synthesis) and Hsepi (epimerase for HS biosynthesis). A pharmacological analysis of its inhibitory activity toward the purified recombinant enzymes showed a strong, yet somewhat similar inhibitory activity toward the three enzymes with an IC_50_ of 7.0 μM for DS-epi, 1.5 μM for D4ST1 and 0.8 μM for Hsepi, respectively (Figure 1B–D). However, ebselen did not inhibit the activity of C4ST1, a 4-*O*-sulfotransferase involved in CS biosynthesis, and the epimerase activity of DS-epi2, a paralog of DS-epi1 (data not shown). Our earlier study showed that DS-epi2 is the predominant epimerase in the brain and has only minor contribution to CS/DS synthesis in other tissues (Maccarana et al. 2009). Hence, ebselen specifically inhibits three enzymes involved in the biosynthesis of iduronic acid in both HS and CS/DS, i.e. the two epimerases HSepi and DS-epi1 and the sulfotransferase D4ST1 which synthesizes the (4-*O*-sulfo-*N*-acetyl-galactosamine-iduronic acid)*_n_* structures.

### Ebselen acts as an irreversible and noncompetitive inhibitor of DS-epi1

**Fig. 2:**
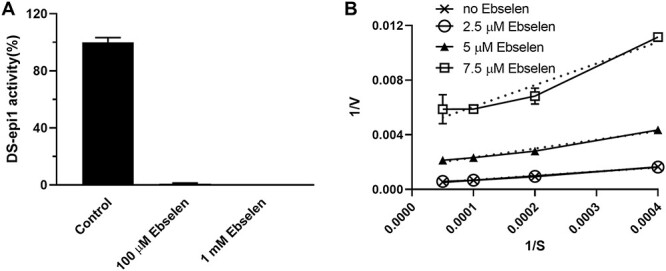
Ebselen has an irreversible, noncompetitive mode of action on DS-epi1. (**A**) Recombinant DS-epi1 (20 ng) was incubated with 100 μM or 1 mM ebselen for 1 h at 37°C. The drug was removed by dialysis against the assay buffer and the DS-epi1 activity was assayed (*n* = 3). (**B**) Kinetic studies by incubation of 2 ng of DS-epi1 with variable amount of the substrate and ebselen for 16 h at 37°C. The data are shown as a Lineaweaver-Burk plot (*n* = 3).

To investigate the inhibitory mechanisms of ebselen, recombinant DS-epi1 was incubated with ebselen (100 μM or 1 mM) for 1 h and was then dialyzed against the enzyme assay buffer. Incubation of the dialyzed enzyme with the substrate detected no epimerase activity at a concentration of 1 mM ebselen and trace amount activity at the concentration of 100 μM (Figure 2A), indicating an irreversible inhibition. A kinetic analysis (Lineaweaver-Burk plot) obtained after incubation with variable concentration of the polysaccharide substrate in the presence of the drug showed a noncompetitive mode of action of ebselen (Figure 2B). The experiments suggest that ebselen is an irreversible and noncompetitive inhibitor of DS-epi1.

### Ebselen treatment reduced iduronic acid content in CS/DS

**Fig. 3:**
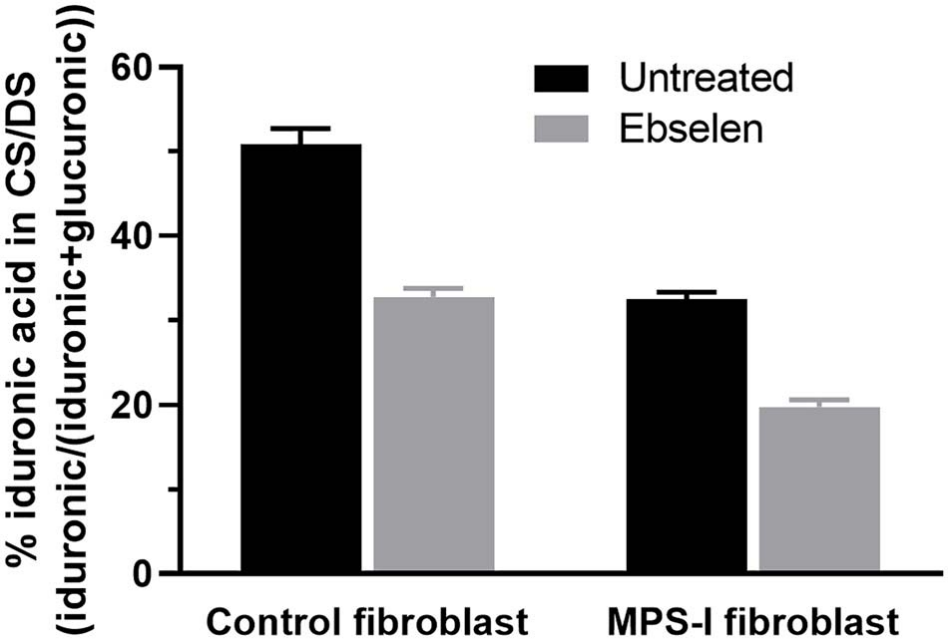
Ebselen reduces iduronic acid formation in CS/DS in control and MPS-I fibroblasts. Cells were cultured in the presence of ebselen (20 μM in control and 40 μM in MPS-I fibroblasts) and ^35^S-sulfate. After 24 h, CS/DS was isolated from the medium and subjected to chondroitinase B digestion which only cleaves the iduronic acid-containing structures. Visualization of the split products on Superdex Peptide gel filtration column allowed the calculation of the content of iduronic acid in CS/DS chains (*n* = 2).

We wondered whether ebselen could inhibit formation of iduronic acid during CS/DS biosynthesis in a cellular context, where a complex biosynthetic machinery is present in the Golgi apparatus. To this aim, we cultured human fibroblasts in the presence of 20 μM ebselen for 24 h, collected the medium and isolated the newly synthesized and released CS/DS. Quantification of iduronic acid was performed by size analysis of the split products obtained after chondroitinase B degradation. A 36% reduction of iduronic acid in CS/DS was found in the ebselen-treated fibroblasts (Figure 3). We next analyzed fibroblasts from an MPS-I patient, which are deficient in the lysosomal L-iduronidase that is responsible for the catabolism of iduronic acid in GAG degradation due to the homozygous mutation p.W402X. Exposure of MPS-I fibroblasts to 40 μM ebselen under the same conditions as described above led to a 39% reduction of iduronic acid in CS/DS (Figure 3). We conclude that ebselen efficiently inhibits iduronic acid production in both control and MPS-I cells.

### Ebselen treatment attenuated GAG accumulation in MPS-I fibroblasts

**Fig. 4:**
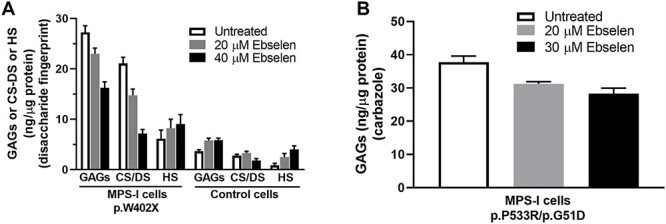
Ebselen decreases overall accumulation of CS/DS in MPS-I fibroblasts. Cells were cultured for 10 days in the presence of ebselen at the concentrations indicated. Medium was changed every second day. The GAGs were purified from the cells. (**A**) CS/DS and HS from control and MPS-I (p.W402X) fibroblasts were completely depolymerized to disaccharides by either chondroitinase ABC or heparinase I, II and III digestion, respectively. The disaccharides were quantified after HPLC separation. The CS/DS and HS content was normalized to the amount of total cellular proteins (*n* = 3). Total GAGs are the sum of CS/DS and HS. (**B**) Total GAGs from MPS-I (p.P533R/p.G651D) fibroblasts were analyzed by the carbazole reaction (*n* = 4).

We further investigated whether ebselen can reduce accumulation of GAGs in MPS-I fibroblasts. The control and MPS-I cells, which have the homozygous mutation p.W402X in the iduronidase gene, were cultured in the presence of ebselen for 10 days, and total GAGs were purified from cells. The quantity of CS/DS and HS was measured by a sensitive disaccharide fingerprint methodology after either chondrotinase ABC or heparinase (I + II + III) digestion (Stachtea et al. 2015). The results showed that untreated MPS-I fibroblasts contained 7- to 8-fold higher total GAGs and CS/DS than control fibroblasts (Figure 4A).

It should be noted that, in both cell types, CS/DS was three times more abundant than HS regardless of the large difference in the total GAG amount between the control and MPS-I fibroblasts. In the ebselen-treated MPS-I fibroblasts, the amounts of total GAGs were reduced in a concentration-dependent manner. At 40 μM of the drug, GAGs were decreased by 40%. A closer look at the distinct GAG subtypes showed that the amounts of CS/DS and HS changed very differently in response to ebselen treatment. While the levels of CS/DS decreased robustly (reduction of 66%), the HS content showed a slight increase. In comparison, no significant CS/DS change was observed upon ebselen treatment in control fibroblasts. Notably, the increase of HS was more pronounced in the control cells, which resulted in an overall minor increase of total GAGs. To ascertain that the effect of ebselen was not limited to MPS-I cells with a defined genotype, a second MPS-I fibroblast cell line, carrying the compound heterozygous mutations p.P533R/p.G51D in the iduronidase gene, was analyzed. Again, total GAGs, analyzed by the carbazole reaction, decreased in a concentration-dependent manner upon ebselen treatment (Figure 4B). Ebselen caused no cell toxicity at the concentrations tested, as evaluated by phenotypic observation and cell proliferation of both cell types. In fact, no difference in the total quantity of recovered cellular proteins was noted between the control and treatment groups of both cell types. Altogether, the data showed the capability of ebselen to decrease the pathological accumulation of CS/DS in MPS-I fibroblasts.

### Ebselen treatment enhanced the catabolism of the GAGs in MPS-I fibroblasts

**Fig. 5:**
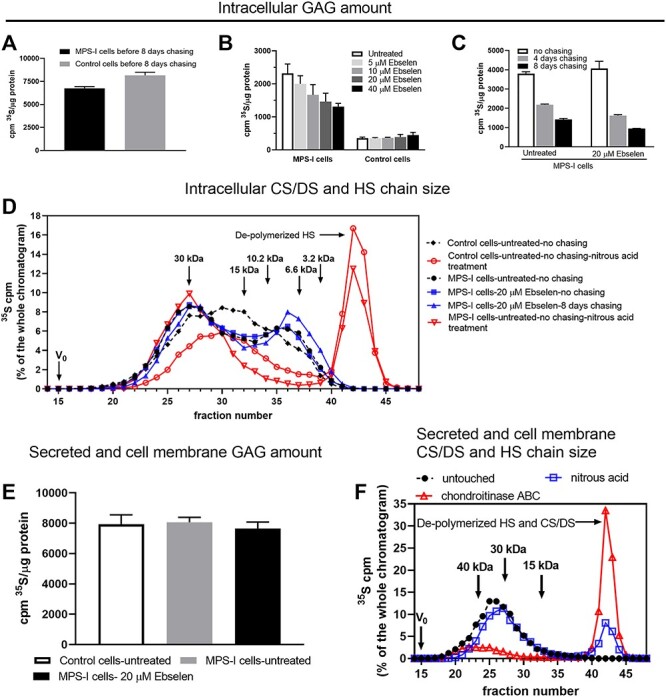
Ebselen accelerates catabolism of GAGs in MPS-I fibroblasts. (**A**–**C**) Effect of ebselen on the intracellular GAG amount. Control and MPS-I (p.W402X) fibroblasts were cultured in the presence of ^35^S-sulfate for 72 h. One aliquot of the cells was collected for GAG isolation and quantification (**A**), and the rest of the cells were equally split in wells of 12-well plates without ^35^S-sulfate for an additional 8 days (chasing) in the presence of ebselen at variable concentrations as indicated (**B**). The medium was changed every second day. After 8 days of chasing, the cells were harvested for GAG isolation and quantification. Radioactivity was normalized by the amount of cellular protein (*n* = 3). (**C**) Time course of the ebselen effect. MPS-I cells were labeled for 3 days either without or with 20 μM ebselen. After the labeling period, the medium was pooled together with the supernatant of the trypsin treatment of cells to represent mostly intact GAGs that are not subjected to endocytosis. Cells were then chased for 4 and 8 days continuing the treatment. Cells were harvested for GAG isolation and quantification. The radioactivity was normalized by the amount of cellular protein (*n* = 3). (**D**) Intracellular CS/DS and HS chain size. GAGs were purified from unchased and 4- and 8-day-chased cells derived from the experiment reported in (**C**) and were applied to a Superose 6 column. *Filled black rhombus*: GAGs from unchased and untreated control cells; *empty red circle*: GAGs from unchased and untreated control cells subjected to nitrous acid deamination at pH 1.5 to depolymerize HS whose degradation products are indicated with an *arrow*; *filled black circle:* GAGs from unchased and untreated MPS-I cells; *filled blue square*: GAGs from unchased and treated with 20 μM ebselen MPS-I cells; *filled blue triangle*: GAGs from 8 days chased and treated with 20 μM ebselen MPS-I cells; *empty red triangle*: GAGs from unchased and untreated MPS-I cells subjected to nitrous acid deamination. The chromatograms from MPS-I cells are basically superimposable irrespective of ebselen treatment and chasing or not chasing conditions (all chromatograms not shown for clarity). (**E**) Production of secreted and cell membrane GAGs. Cells were labeled for 3 days as in (**C**) and then medium was collected, cells were trypsinized and the supernatant after cell trypsinization was pooled with medium. GAGs were purified and normalized by cell protein (*n* = 3). (**F**) Secreted and cell membrane CS/DS and HS chain size. GAGs, prepared as described in (**E**), were size-fractionated on a Superose 6 column before (*black filled circle*), or after nitrous acid degradation (*empty blue square*), or after chondroitinase ABC degradation (*empty red triangle*). Shown are the chromatograms of untreated MPS-I cells, but the ones derived from untreated control cells or 20 μM ebselen-treated MPS-I cells are superimposable (not shown for clarity).

The result above showed that total GAGs was lowered upon ebselen treatment in MPS-I fibroblasts. In order to investigate the mechanism(s) involved, we applied a pulse-chase radiolabeling technique. The cells were cultured in the presence of ^35^S-sulfate for 3 days. One aliquot of the cells was collected at the end of the labeling, and one aliquot was washed and split into 12-well plates in the culture medium without ^35^S-sulfate. After 8 days of chasing in the presence of ebselen at the concentrations of 5–40 μM (medium changed every second day), cells were collected for GAG isolation. Quantification of the total ^35^S-sulfate-GAGs isolated from the cells collected at the end of the labeling showed slightly less labeled GAGs in MSP-I cell when compared to the control cells (Figure 5A). Quantification of the GAGs after chasing showed a dramatically reduced amount of the ^35^S-sulfated-GAGs in the control cells from 8000 dpm to 400 dpm per μg of total protein (Figure 5B). In comparison, the labeled GAGs in the MPS-I cell were reduced from 6500 dpm to 2200 dpm per μg of total protein (Figure 5B). Ebselen treatment had no apparent effect on the control cells but resulted in a concentration-dependent reduction of labeled GAGs in the MPS-I cells with a 41% decrease at a concentration of 40 μM when compared to untreated cells. These data suggest that ebselen promotes the catabolism of GAGs accumulated in MPS-I fibroblast. Next, the kinetic of ebselen-dependent reduction of intracellular GAGs was examined. MPS-I cells were ^35^S-sulfate labeled for 3 days in the absence or presence of 20 μM ebselen, differently from the experiment described in Figure 5A and 5B, and then chased (medium without ^35^S-sulfate) for 4–8 days in the presence of the drug. There was no difference in the amount of incorporated GAG between untreated and ebselen-treated cells at the end of labeling (Figure 5C), but the effect of ebselen on lowering GAG levels became apparent already after 4 days of chasing. Next, we examined which GAG molecular species were accumulating in MPS-I cells and which ones were decreased upon ebselen treatment. The molecular size of labeled GAGs was analyzed by gel permeation chromatography (Figure 5D). In untreated MPS-I cells, two defined peaks were present after 3 days of labeling, one around ~30 kDa constituted of CS/DS, and the second appeared around ~8 kDa, representing HS, which is in good agreement with the lysosomal HS length found in MPS-IIIa fibroblasts (Lamanna et al. 2012). The properties of CS/DS versus HS were confirmed by nitrous acid treatment at pH 1.5, which is a chemical depolymerization specific to HS. CS/DS represented 65% of the total labeled GAGs, which is in agreement with the predominance of CS/DS over HS in the steady-state situation (Figure 4A). Both CS/DS and HS chains did not change in terms of length and ratio upon ebselen treatment before and after chasing (Figure 5D shows the chromatogram of untreated cells after 8 days of chasing, but all chromatograms, irrespective of treatment and chasing time, are basically superimposable [not shown for clarity]). By comparison, the control fibroblasts, after 3 days of labeling, showed an overlap of labeled CS/DS and HS approximately from 30 kDa to 8 kDa, indicating a normal degradation of both species (Figure 5D). In summary, newly synthesized CS/DS and HS chains with a length of ~30 kDa and ~8 kDa, respectively, accumulated in the MPS-I lysosomes, while ebselen treatment proportionally reduced the amounts but not the sizes of these GAGs.

### Ebselen treatment does not alter GAG production in MPS-I fibroblasts

One mechanism of ebselen action on MPS-I fibroblast could be that the drug might reduce the biogenesis selectively of either CS/DS or HS or of both GAGs. To measure GAGs which were not, or minimally, subjected to internalization, the medium produced by the cells, described in Figure 5C, was pooled with the cell membrane GAGs. The amounts of GAGs from untreated control cells, untreated MPS-I cells and MPS-I cells treated with 20 μM ebselen did not significantly differ (Figure 5E). In untreated MPS-I cells, CS/DS chains, representing 77% of the total GAGs, peaked around ~30 kDa, and HS peaked around ~43 kDa (Figure 5F). The chromatograms derived from control fibroblasts or ebselen-treated MPS-I cells were superimposable to the ones derived from untreated MPS-I cells (data not shown for clarity). In summary, neither the production nor the length of CS/DS and HS changed after ebselen treatment and it did not vary between control and MPS-I fibroblasts.

### Ebselen treatment mimics DS-epi1 loss of function and blocks NC cell migration in *Xenopus* embryos

**Fig. 6:**
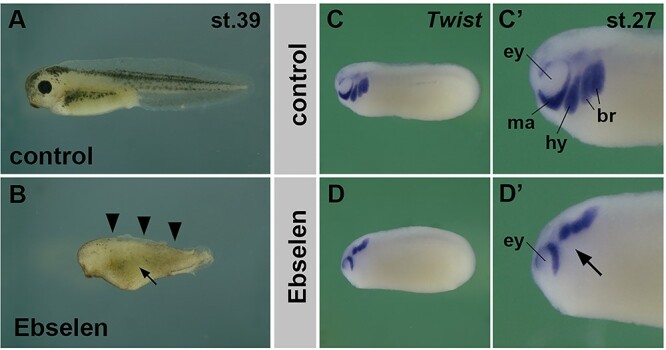
Ebselen blocks NC cell migration in *Xenopus* embryos, phenocopying the effect of Dse/DS-epi1 knockdown. (**A**) Control *Xenopus* embryo at tadpole stage. (**B**) Sibling embryo after treatment with 12.5 μM ebselen. Note the reduction of head and eye structures, loss of dorsal fin structures (arrowheads) and low number of pigmented melanocytes (arrow), which suggest defects in NC cell development. (**C** and **C′**) Early tailbud embryo in side view after whole-mount in situ hybridization. *Twist* (blue stain) demarcates four distinct streams of cranial NC cells which migrate ventrally in the developing head: mandibular cells ventral to the eye, followed by hyoid and two branchial arch cell streams more posteriorly. (**D** and **D′**) Ebselen treatment suppresses the migration of cranial NC cells. Note that *Twist*^+^ cells remain dorsal to the eye and in the dorsal head (arrow). The proportion of embryos with the described phenotypes was as follows: A, 43/43; B, 25/25 (small head), 20/25 (reduced dorsal fin), 24/25 (less melanocytes); C, 60/60; D, 53/53. Each experiment was at least performed two times. br, branchial arch; ey, eye; hy, hyoid arch; ma, mandibular arch.

We previously showed that knockdown of Dse/DS-epi1 causes a reduction of iduronic acid in CS/DS of early *Xenopus* embryos (Gouignard et al. 2016). Therefore, we first used this vertebrate model to investigate the role of ebselen in vivo (Figure 6). Exposure to ebselen at a concentration of 12.5 μM from the neurula stage onward caused reduction of head and eye structures, a decrease of melanocytes and loss of dorsal fin tissue in *Xenopus* tadpole embryos (Figure 6A and B). NC cells not only contribute to cartilage and soft tissue in the head and give rise to melanocytes (Mayor and Theveneau 2013), but they also induce and form the dorsal fin (Tucker and Slack 2004). We therefore hypothesized that ebselen might inhibit NC development. Whole-mount in situ hybridization with an antisense RNA probe against *Twist* demarcates cranial neural crest (CNC) cells migrating ventrally in four distinct streams (mandibular, hyoid, anterior and posterior branchial arch) in the head of embryos at the tailbud stage (Figure 6C and C’). Of note, ebselen did not affect the formation of *Twist*-expressing CNC cells but inhibited their ventralward migration (Figure 6D and D’). Ebselen caused mandibular CNC cells to stay dorsal to the eye anlage and prevented hyoid and branchial arch CNC cells from leaving their site of origin at the neural plate border. This effect of ebselen treatment is strikingly similar to the phenotype caused by knockdown of Dse (Supplementary Figure S1) and (Gouignard et al. 2016). *Xenopus Dse* and *Dsel*, the latter coding for DS-epi2, exhibit distinct expression domains in the early embryo, including the developing NC (Gouignard et al. 2018). The phenotypic resemblance of ebselen treatment and Dse–MO injection underscore the important function of DS-epi1 in NC development. The observation that both the drug and Dse knockdown cause defects in CNC cell migration suggests that ebselen acts as an inhibitor of DS epimerase activity in the *Xenopus* embryo.

### Ebselen does not decrease GAGs or CS/DS accumulation in the MPS-I mouse model

**Fig. 7:**
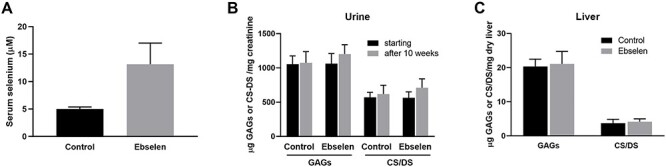
Ebselen does not change CS/DS and GAG content in urine and organs of MPS-I mice. Five MPS-I mice were fed with food supplemented with ebselen to give an intake of 100 mg/kg/day/mouse and five MPS-I mice were fed with control food for 10 weeks. At the end of the treatment period, serum ebselen, measured as selenium, was quantified by inductively coupled plasma mass spectrometry (**A**) (*n* = 4). GAGs were purified from urine and organs and quantified by the carbazole reaction (analytical triplicates). The CS/DS was separately quantified by disaccharide fingerprint following chondroitinase ABC digestion (analytical duplicates). Urine is shown in (**B**) (*n* = 5) and liver in (**C**) (*n* = 5). Brain, kidney and spleen are shown in Supplementary Figure S2.

Given the promising activity of ebselen on MPS-I fibroblasts and its effect on *Xenopus* embryological development, we tested the compound on the MPS-I mice, a commonly used animal model of the human disease (Clarke et al. 1997). Ebselen was incorporated in grinded pellet to reach an intake of 100 mg/kg/day/mouse. Five MPS-I mice were fed with ebselen-enriched food and five mice with control grinded pellet, with refreshment of the food every second day for 10 weeks. At the end of the treatment, the serum level of ebselen was 8.2 μM, calculated by the difference of selenium between treated and untreated mice (Figure 7A). Daily monitoring did not reveal weight loss or other phenotypic deviations between the treated and control mice. Quantification of the total GAGs and CS/DS in the urine and selected organs did not show any difference between the treated and control group (Figure 7B and C and Supplementary Figure S2). Interestingly, CS/DS showed a tendency to increase in the urine. For a functional evaluation, during three consecutive days before termination of the treatment period, mice were tested for general locomotory activity in an open field test. Three out of five treated mice showed hyper-activity. The frequency of line crossing in the open field of these three mice was two to three times higher than the average of the other animals included in the study (data not shown). In summary, ebselen failed to decrease the pathological accumulation of CS/DS and GAGs in the MPS-I mouse model.

## Discussion

Since the MPS-I syndrome is caused by a genetic defect in L-iduronidase which removes iduronic acid from the GAG chains of CS/DS and HS (Clarke et al. 1997), we hypothesized that a reduction of iduronic acid formation may decrease pathological accumulation of the GAGs, leading to a therapeutic approach of substrate reduction therapy (SRT). SRT is in clinical use for other lysosomal storage disorders (Coutinho et al. 2016) and has been validated in a mouse model of MPS-IIIa by reduction of HS biosynthesis (Lamanna et al. 2012). The use of rhodamine B, which decreases GAG biosynthesis by an unspecific mechanism, produced limited benefit for MPS-I mice (Derrick-Roberts et al. 2017). Prompted by these studies, we selected DS-epi1 as the primary target to reduce the formation of iduronic acid in CS/DS biosynthesis (Malmstrom et al. 2012). As an initial screening, we chose the Prestwick library because it is composed of drugs and prospective drugs, which should facilitate follow-up studies with a repurposing strategy. Having seen a strong inhibitory effect of ebselen on DS-epi1, we wanted to know whether this drug also affects the activity of other enzymes involved in iduronic acid synthesis in GAGs. Interestingly, ebselen demonstrated a comparable inhibitory activity toward the enzymes involved in iduronic acid biosynthesis both in CS/DS, DS-epi1 and D4ST1 and in HS, Hsepi. On the other hand, the drug did not inhibit the CS/DS biosynthetic enzyme DS-epi2 and C4ST1, which is a 4-*O*-sulfotransferase involved in CS biosynthesis. This may imply a cumulative and selective activity of ebselen on iduronic acid biosynthesis since DS-epi1 and D4ST1 are closely interrelated (Tykesson et al. 2018) and Hsepi has a similar catalytic mechanism as DS-epi1 (Hagner-Mcwhirter et al. 2000; Hasan et al. 2021).

We focused on DS-epi1 and CS/DS biosynthesis because we found that fibroblasts from both healthy donor and MPS-I patients contain three times more CS/DS than HS, which may suggest a greater contribution of CS/DS to the GAG accumulation in MPS-I fibroblasts. Importantly, ebselen inhibited iduronic acid production in CS/DS also in a cellular context. Regarding the mechanism of action, ebselen has an irreversible and noncompetitive activity on DS-epi1, likely by covalent binding to thiol groups in cysteine(s), as it has been demonstrated with many other proteins (Azad and Tomar 2014). The Golgi luminal part of human DS-epi1 contains six cysteines, which are all present in the recently solved crystal structure of human DS-epi1 (Hasan et al. 2021). Interestingly, the only solvent-accessible residues, Cys 494 and 506, are both located at the same position (only 3.5 Å apart), adjacent to the active-site groove, where ebselen modification could potentially alter the catalytic activity of DS-epi1.

With the above data as background, we tested ebselen to reduce the pathological accumulation of CS/DS and HS in MPS-I fibroblasts. The results showed a concentration-dependent decrease in CS/DS and a minor and contrasting effect on HS. This effect was tested in two MPS-I cells carrying different mutations in the iduronidase gene, demonstrating a general effect of ebselen (Figure 4). The molecular mechanism through which MPSs cells decrease accumulation of GAGs upon a substrate reduction treatment is debated (Banecka-Majkutewicz et al. 2012). Ebselen altered neither the amount nor the size of secreted GAGs after 3 days of labeling (Figure 5E and F). This fraction contained a majority of CS/DS chains distributed around 30 kDa and HS chains distributed around 43 kDa. On the other hand, ebselen decreased the amount but not the size of the intracellular chains after 3 days of labeling with or without chasing for 4 and 8 days (Figure 5A–D). The results showed that the size of CS/DS was not changed (30 kDa) and the size of HS was significantly decreased to approximately 8 kDa, which is similar to the size of lysosomal MPS-IIIa HS chains (Lamanna et al. 2012). These results may imply that GAGs are degraded, with the help of residual iduronidase activity or by other mechanisms (Banecka-Majkutewicz et al. 2012), in a processive way, and a possible compensation by heparanase, the endoglucuronidase specific to HS (Pikas et al. 1998). The absence of effect of ebselen on CS/DS chain length is in agreement with the observation in DS-epi1 mutant mice (Maccarana et al. 2009) or DS-epi1 downregulation in cells (Bartolini et al. 2013). In summary, ebselen promoted catabolism of the GAGs in MPS-I fibroblast. Meanwhile, it is worth to note that even in the control fibroblasts, some labeled GAGs are still present after 8 days of chasing. It has been proposed that the GAGs have a rapid turnover in cells, with a half-life of less than 24 h (Owens and Wagner 1991). It will be interesting to characterize these GAGs with regard to molecular structure and cellular localization.

Encouraged by its activity in vitro, ebselen was tested in vivo in early frog development and in an MPS-I mouse model. In *Xenopus* embryos, ebselen treatment caused suppression of cranial NC cell migration and a loss of NC-derived melanocytes and dorsal fin structures, which is in striking similarity with the effects caused by DS-epi1 downregulation (Gouignard et al. 2016), suggesting that the drug inhibits DS-epi1 in vivo. However, albeit that ebselen has displayed satisfactory effectivity in inhibition of iduronic acid formation and on GAG accumulation in the MPS-I cells, the drug did not show any apparent effect in MPS-I mice. We may assume that the treatment strategy was not optimal, e.g. route of administration, dosage and duration, although at the end of the treatment, the ebselen serum concentration was 8 μM which is higher than the IC_50_ for DS-epi1, D4ST1 and HSepi. Nonetheless, the results confirmed previous data of good oral absorption, high bioavailability and low toxicity (Kil et al. 2017). Our results raise questions about the use of ebselen, specially for a long-term treatment, given its multi-target spectrum.

In summary, our study demonstrated ebselen as an efficient DS-epi1 inhibitor that can inhibit formation of iduronic acid in CS/DS biosynthesis and cam reduce GAG accumulation in MPS-I cells. Even if ebselen failed to show any effect on mice in our experimental conditions, the study provided novel information to develop strategies for treatment of MPS-I by downregulating iduronic acid biosynthesis and more generally in other MPSs by enhancing GAG catabolism. Moreover, the finding that ebselen inhibits the activities of DS-epi1, D4ST1 and Hsepi also offers a valuable tool for studies on the properties of these enzymes and their biological functions in different settings.

## Materials and methods

### Reagents and analytical tools

Ebselen was purchased from Sigma and 50 mM stock solution was prepared in DMSO. ^35^S-sodium sulfate (1500 Ci/mmol) was from PerkinElmer. Sulfate-free Dulbecco’s modified Eagle’s medium (DMEM) (AS31600 cat no. 074-91083P) was from Gibco. Superose 6 10/300, Superdex Peptide 10/300, PD-10 columns, Sephadex G-25, DEAE-Sephacel were from Cytiva.

### Recombinant enzymes

To screen the Prestwick library, recombinant DS-epi1 was purified from the supernatant of HEK293 cells that were transiently transfected with the full-size (amino acid 1–958) human DS-epi1-myc-HIS sequence inserted in the pcDNA.3 expression vector (Maccarana et al. 2006). The medium (serum-free) was concentrated with Centriprep Centrifugal Filter Units MWCO 30 kDa (Millipore) and buffer-exchanged to the DS-epi assay buffer by PD-10 column. For all tests following the primary screening, the truncated form (amino acid 23–775-HIS) recombinant human DS-epi1 was prepared as described (Tykesson et al. 2016). Recombinant human D4ST1-HIS (amino acid 61–376) was produced as described (Tykesson et al. 2018). Recombinant full-length human HSepi (GLCE) was cloned into pPICZa expression vector (Invitrogen) and was produced as fusion protein-myc-HIS in the medium of *Pichia pastoris* cultivation. The media was concentrated and dialyzed into assay buffer.

### Enzyme activity assays

The activity of DS-epi1 was assayed in 100 μL of 20 mM MES, pH 5.5, 10% glycerol, 2 mM MnCl2, using 30,000 dpm of the substrate [5-^3^H]dK4 (preparation of the substrate according to Hannesson et al. 1996 and assay adapted from Maccarana et al. 2006). After 16–20-h incubation at 37°C, 90 μL of the incubation mixture was added to a scintillation vial containing 5 mL of biphasic scintillation cocktail (three volumes of INSTA-FLUOR PLUS [Perkin Elmer 6013167] + one volume of isoamylalcohol) (Campbell et al. 1994). The vials were vortexed for 30 s and equilibrated for at least 6 h before scintillation counting for radioactivity. Background was ≤200 dpm.

D4ST1 was assayed in 50 μL of 50 mM imidazole-HCl pH 6.8, 10^6^ dpm [^35^S]PAPS, O-desulfated re-N-sulfated heparin as acceptor polysaccharide (10 nmol as glucuronic acid) (Mikami et al. 2003). The reaction mixtures were incubated at 37°C for 2–4 h and subjected to gel filtration using a 2-mL syringe column packed with Sephadex G-25 (superfine) made dry by centrifugation. [^35^S]-sulfate incorporation into polysaccharides was quantified by the determination of the radioactivity in the flow-through fractions by liquid scintillation counting (Wlad et al. 1994). HSepi was assayed in 50 μL of 25 mM Hepes pH 7.0, BSA 100 μg/mL, 100 mM KCl, 1 mM CaCl2, using 30,000 dpm of [5-^3^H]-N-sulfated K5 polysaccharide as substrate (assay modified from Campbell et al. 1994 and substrate produced according to Hagner-Mcwhirter et al. 2000 with one modification, i.e. deacetylation was not performed by hydrazine treatment but by alkaline treatment [incubation of the labeled K5 with 2 M NaOH at 60°C for 16 h]). After 16–20-h incubation at 37°C, 45 μL of the incubation mixture was mixed with 5 mL of the biphasic scintillation cocktail, and counted, as described for the DS-epi1 assay.

### Screening of the Prestwick library

The Prestwick library (1200 drug compounds) was provided by Chemical Biology Consortium Sweden as 50 mM stock compounds in DMSO, and 100 nl was spotted in 96-well microtiter plates. The final concentration of the compounds was 50 μM in the volume of 100 μL assay mixture for DS-epi1.

### Cell cultures

Primary human skin fibroblasts, derived from 1-year-old MPS-I patients and a healthy age-matched donor, were obtained from the “Cell line and DNA Biobank from Patients affected by Genetic Diseases” (Institute G. Gaslini, Genova, Italy). MPS-I fibroblasts had a stop codon on both alleles of the iduronidase gene, causing a truncated protein p.W402X, which is the most common MPS-I variants (31% worldwide) (Kubaski et al. 2020). Analysis of the fibroblast lysate using the fluorogenic substrate 4-methylumbelliferyl-α-L-iduronide (Ou et al. 2014) confirmed significantly reduced iduronidase activity (5000 times less in p.W402X MPS-I when compared to control fibroblasts). The MPS-I fibroblasts, used only in Figure 4B, had the compound heterozygote iduronidase mutation p.P533R/p.G51D. Fibroblasts were maintained in DMEM, 10% FBS and 100 units/mL penicillin and 100 μg/mL streptomycin.

### Quantification of iduronic acid content in CS/DS

The analysis was conducted as in Stachtea et al. (2015) with modifications. The cells were plated in DMEM medium and the day after, at a confluency of 90%, changed to sulfate-free DMEM, 10% FBS, 10 units/mL penicillin and 10 μg/mL streptomycin, with the addition of 100 μCi/mL ^35^S-sulfate and ebselen. After 24 h, the medium was recovered for purification of proteoglycans (PGs) by DEAE-column chromatography in the presence of 6 M urea. The eluted PGs were desalted and subjected to pronase digestion followed by deaminative cleavage of HS at pH 1.5. CS/DS was re-isolated on Superose 6 and the degraded HS disaccharides were removed. The purity of CS/DS was verified by analytical digestion with chondroitinase ABC (Sigma C3667). CS/DS was degraded by chondroitinase B, which only cleaves the iduronic acid~*N*-acetyl galactosamine linkages, (R&D System; 2 mIU/incubation) in 20 mM Hepes, pH 7.2, 50 mM NaCl, 4 mM CaCl_2_ and 0.1 mg/mL BSA for 2 h at 37°C. The split products were separated on a Superdex Peptide column and the percentage of iduronic acid over the total iduronic acid + glucuronic acid was calculated.

### Quantification of unlabeled cellular GAGs

Control and MPS-I fibroblasts were cultured in 12 wells, starting from 10% confluency, in the presence of ebselen, and the medium was changed every second day, for 10 days. Then the cells were trypsinized, washed with PBS and the cell pellet was lysed in 100 μL of 20 mM MES pH 6.5, 150 mM NaCl and 0.1% Triton. After centrifugation, the supernatant was analyzed for protein concentration by the Bradford assay (Bio-Rad), and the GAGs were purified and quantified according to Stachtea et al. (2015). Briefly, proteins were degraded by pronase and DNA by DNAase and GAGs were purified by DEAE. GAGs were quantified by carbazole reaction (Figure 4B) or, alternatively, as shown in Figure 4A, CS/DS and HS were quantitatively digested to disaccharides, either by digestion with chondroitinase ABC (Sigma C3667) or with a mixture of heparinase I plus II plus III, respectively (heparinases were in-house preparations, purified from *Escherichia coli* transformed with the pET-15b expression vector carrying heparinase I, or with pET-19b expression vector carrying heparinase II or III; bacteria were provided by Prof. Jian Liu, University of North Carolina). Disaccharides were fluorescently labeled by 2-aminoacridone and separated by HPLC. CS/DS or HS quantification was obtained summing up the amount of all known disaccharide peaks when compared to the peaks generated by known weight of standard disaccharides (Iduron, UK).

### Quantification and size-fractionation of 35S-labeled GAGs obtained after pulse-chase experiment

Control and MPS-I fibroblasts in T75 flask were cultured in the presence of ^35^S-sulfate (100 μCi/mL) in sulfate-free DMEM, 10% FBS, 10 units/mL penicillin and 10 μg/mL streptomycin for 72 h. Then, one aliquot of the cells was collected for purification of GAGs, and the rest of the cells were split into 12-well plates in DMEM medium (without ^35^S-sulfate) containing variable concentrations of ebselen. Ebselen-containing and control medium were changed every second day. After 8 days of chasing, the cells were trypsinized, washed with PBS and the collected pellet was lysed in 100 μL 20 mM MES pH 6.5, 150 mM NaCl, 0.1% Triton. Protein was quantified by the Bradford assay (Bio-Rad) and the ^35^S-sulfate-labeled GAGs, detached from the core protein by beta-elimination (50 mM KOH, 0.1 M NaBH_4_, 16 h, 45°C), were purified by DEAE. In the experiment which is described in Figure 5C–F, 20 μM ebselen was present also in the labeling period. GAG chains were applied to Superose 6 column, run in 0.2 M ammonium bicarbonate at 0.25 mL/min and fractions were collected every 2 min. Peak elution volumes were assessed using size-defined GAGs as previously described and as indicated in the chromatograms (Petersen et al. 1999).

### *Xenopus* embryo manipulations

All *Xenopus* experiments reported in this study have been approved by the Lund/Malmö regional ethical committee (M140-14 to E.M.P). Embryos were prepared, cultured and analyzed by Red-Gal staining for lineage tracing and whole-mount in situ hybridization as described (Pera et al. 2015).

For the pharmacological inhibitor treatment, embryos were incubated from neurula stage (stage 14) onward in a 24-well plate (10–15 embryos per well) at 17°C in 0.1× Modified Barth’s Saline (MBS), 0.1% BSA, 0.025% dimethyl sulfoxide (DMSO) alone as control or together with 12.5 μM ebselen.

### MPS-I mice treatment with ebselen

MPS-I mice, deficient in the L-iduronidase gene, were obtained from IRCCS San Raffaele, Milano, Italy, and were described in Clarke (2008). The experiments were initiated with 14-week-old mice. Daily ebselen injections were not allowed by the ethical committee and therefore ebselen was incorporated into grinded pellet food as an ethanol solution. Ethanol was then evaporated. In preliminary experiments, it was determined how many grams of grinded pellet were consumed every 2 days, and the amount of ebselen was adjusted to have an intake of 100 mg/kg/day/mouse. For 10 weeks, five MPS-I mice were fed with ebselen-enriched grinded pellet and five mice were fed with ethanol-control grinded pellet. Food was substituted every second day. Experiments were approved by the Ethical Committee of Lund University (M19-16 to M.M.) according to the Swedish law and national guidelines. At the end of treatment, serum ebselen, a selenium-containing compound, was measured as selenium by inductively coupled plasma mass spectrometry by the Clinical Chemistry Department of the Gothenburg hospital. Serum creatinine was measured by the Clinical Chemistry Department of Lund hospital. GAGs were purified from organs and urine and CS/DS was measured according to Stachtea et al. (2015). Total GAGs were measured by the carbazole reaction.

## Authors’ contributions

M.M., E.T., E.M.P., N.G., J.F., A.M., G.G. and J.-p.L. contributed to the collection, analysis and interpretation of data. M.M., E.M.P. and J.-p.L. wrote the manuscript. All authors read and approved the final manuscript.

## Supplementary Material

20210608_Supplementary_data_resubmitted_cwab066

Fig_S1_resubmitted_cwab066

Fig_S2_resubmitted_cwab066

## References

[ref1] Azad GK, Tomar RS. 2014. Ebselen, a promising antioxidant drug: mechanisms of action and targets of biological pathways. Mol Biol Rep. 41:4865–4879. 24867080 10.1007/s11033-014-3417-x

[ref2] Banecka-Majkutewicz Z, Jakobkiewicz-Banecka J, Gabig-Ciminska M, Wegrzyn A, Wegrzyn G. 2012. Putative biological mechanisms of efficiency of substrate reduction therapies for mucopolysaccharidoses. Arch Immunol Ther Exp (Warsz). 60:461–468. 22949095 10.1007/s00005-012-0195-9

[ref3] Bartolini B, Thelin MA, Svensson L, Ghiselli G, van Kuppevelt TH, Malmstrom A, Maccarana M. 2013. Iduronic acid in chondroitin/dermatan sulfate affects directional migration of aortic smooth muscle cells. PLoS One. 8:e66704. 23843960 10.1371/journal.pone.0066704 PMC3699603

[ref4] Campbell P, Hannesson HH, Sandback D, Roden L, Lindahl U, Li JP. 1994. Biosynthesis of heparin/heparan sulfate. Purification of the D-glucuronyl C-5 epimerase from bovine liver. J Biol Chem. 269:26953–26958. 7929434

[ref5] Chen HH, Sawamoto K, Mason RW, Kobayashi H, Yamaguchi S, Suzuki Y, Orii K, Orii T, Tomatsu S. 2019. Enzyme replacement therapy for mucopolysaccharidoses; past, present, and future. J Hum Genet. 64:1153–1171. 31455839 10.1038/s10038-019-0662-9

[ref6] Clarke LA . 2008. The mucopolysaccharidoses: A success of molecular medicine. Expert Rev Mol Med. 10:e1. 18201392 10.1017/S1462399408000550

[ref7] Clarke LA, Russell CS, Pownall S, Warrington CL, Borowski A, Dimmick JE, Toone J, Jirik FR. 1997. Murine mucopolysaccharidosis type I: targeted disruption of the murine alpha-L-iduronidase gene. Hum Mol Genet. 6:503–511. 9097952 10.1093/hmg/6.4.503

[ref8] Coutinho MF, Santos JI, Alves S. 2016. Less is more: Substrate reduction therapy for lysosomal storage disorders. Int J Mol Sci. 17:1065. 10.3390/ijms17071065 PMC4964441 27384562

[ref9] Derrick-Roberts ALK, Jackson MR, Pyragius CE, Byers S. 2017. Substrate deprivation therapy to reduce glycosaminoglycan synthesis improves aspects of neurological and skeletal pathology in MPS I mice. Diseases. 5:5. 10.3390/diseases5010005 PMC5456338 28933358

[ref10] Gouignard N, Maccarana M, Strate I, von Stedingk K, Malmstrom A, Pera EM. 2016. Musculocontractural Ehlers-Danlos syndrome and neurocristopathies: Dermatan sulfate is required for *Xenopus* neural crest cells to migrate and adhere to fibronectin. Dis Model Mech. 9:607–620. 27101845 10.1242/dmm.024661 PMC4920151

[ref11] Gouignard N, Schon T, Holmgren C, Strate I, Tasoz E, Wetzel F, Maccarana M, Pera EM. 2018. Gene expression of the two developmentally regulated dermatan sulfate epimerases in the *Xenopus* embryo. PLoS One. 13:e0191751. 29370293 10.1371/journal.pone.0191751 PMC5784981

[ref12] Hagner-Mcwhirter A, Lindahl U, Li JP. 2000. Biosynthesis of heparin/heparan sulphate: mechanism of epimerization of glucuronyl C-5. Biochem J. 347(1):69–75. 10727403 PMC1220932

[ref13] Hampe CS, Eisengart JB, Lund TC, Orchard PJ, Swietlicka M, Wesley J, McIvor RS. 2020. Mucopolysaccharidosis type I: A review of the natural history and molecular pathology. Cell. 9:1838. 10.3390/cells9081838 PMC7463646 32764324

[ref14] Hannesson HH, Hagner-McWhirter A, Tiedemann K, Lindahl U, Malmstrom A. 1996. Biosynthesis of dermatan sulphate. Defructosylated Escherichia coli K4 capsular polysaccharide as a substrate for the D-glucuronyl C-5 epimerase, and an indication of a two-base reaction mechanism. Biochem J. 313(2):589–596. 8573097 10.1042/bj3130589 PMC1216948

[ref15] Hasan M, Khakzad H, Happonen L, Sundin A, Unge J, Mueller U, Malmström J, Westergren-Thorsson G, Malmström L, Ellervik U, et al. 2021. The structure of human dermatan sulfate epimerase 1 emphasizes the importance of C5-epimerization of glucuronic acid in higher organisms. Chem Sci. 12(5):1869–1885. 33815739 10.1039/d0sc05971d PMC8006597

[ref16] Kil J, Lobarinas E, Spankovich C, Griffiths SK, Antonelli PJ, Lynch ED, Le Prell CG. 2017. Safety and efficacy of ebselen for the prevention of noise-induced hearing loss: A randomised, double-blind, placebo-controlled, phase 2 trial. Lancet. 390:969–979. 28716314 10.1016/S0140-6736(17)31791-9

[ref17] Kosho T . 2016. CHST14/D4ST1 deficiency: New form of Ehlers-Danlos syndrome. Pediatr Int. 58:88–99. 26646600 10.1111/ped.12878

[ref18] Kubaski F, de Oliveira Poswar F, Michelin-Tirelli K, Matte UDS, Horovitz DD, Barth AL, Baldo G, Vairo F, Giugliani R. 2020. Mucopolysaccharidosis type I. Diagnostics (Basel). 10:1–23. 10.3390/diagnostics10030161 PMC7151028 32188113

[ref19] Lamanna WC, Lawrence R, Sarrazin S, Lameda-Diaz C, Gordts PL, Moremen KW, Esko JD. 2012. A genetic model of substrate reduction therapy for mucopolysaccharidosis. J Biol Chem. 287:36283–36290. 22952226 10.1074/jbc.M112.403360 PMC3476295

[ref20] Maccarana M, Kalamajski S, Kongsgaard M, Magnusson SP, Oldberg A, Malmstrom A. 2009. Dermatan sulfate epimerase 1-deficient mice have reduced content and changed distribution of iduronic acids in dermatan sulfate and an altered collagen structure in skin. Mol Cell Biol. 29:5517–5528. 19687302 10.1128/MCB.00430-09 PMC2756890

[ref21] Maccarana M, Olander B, Malmstrom J, Tiedemann K, Aebersold R, Lindahl U, Li JP, Malmstrom A. 2006. Biosynthesis of dermatan sulfate: Chondroitin-glucuronate C5-epimerase is identical to SART2. J Biol Chem. 281:11560–11568. 16505484 10.1074/jbc.M513373200

[ref22] Malmstrom A, Bartolini B, Thelin MA, Pacheco B, Maccarana M. 2012. Iduronic acid in chondroitin/dermatan sulfate: Biosynthesis and biological function. J Histochem Cytochem. 60:916–925. 22899863 10.1369/0022155412459857 PMC3527884

[ref23] Mayor R, Theveneau E. 2013. The neural crest. Development. 140:2247–2251. 23674598 10.1242/dev.091751

[ref24] Mikami T, Mizumoto S, Kago N, Kitagawa H, Sugahara K. 2003. Specificities of three distinct human chondroitin/dermatan N-acetylgalactosamine 4-O-sulfotransferases demonstrated using partially desulfated dermatan sulfate as an acceptor: Implication of differential roles in dermatan sulfate biosynthesis. J Biol Chem. 278:36115–36127. 12847091 10.1074/jbc.M306044200

[ref25] Ou L, Herzog TL, Wilmot CM, Whitley CB. 2014. Standardization of alpha-L-iduronidase enzyme assay with Michaelis-Menten kinetics. Mol Genet Metab. 111:113–115. 24332804 10.1016/j.ymgme.2013.11.009 PMC4014300

[ref26] Owens RT, Wagner WD. 1991. Metabolism and turnover of cell surface-associated heparan sulfate proteoglycan and chondroitin sulfate proteoglycan in normal and cholesterol-enriched macrophages. Arterioscler Thromb. 11:1752–1758. 1931877 10.1161/01.atv.11.6.1752

[ref27] Pera EM, Acosta H, Gouignard N, Climent M. 2015. Whole-mount in situ hybridization and immunohistochemistry in *Xenopu*s embryos. In Situ Hybridization Methods. 99:151–167.

[ref28] Petersen F, Brandt E, Lindahl U, Spillmann D. 1999. Characterization of a neutrophil cell surface glycosaminoglycan that mediates binding of platelet factor 4. J Biol Chem. 274:12376–12382. 10212210 10.1074/jbc.274.18.12376

[ref29] Pikas DS, Li JP, Vlodavsky I, Lindahl U. 1998. Substrate specificity of heparanases from human hepatoma and platelets. J Biol Chem. 273:18770–18777. 9668050 10.1074/jbc.273.30.18770

[ref30] Prechoux A, Halimi C, Simorre JP, Lortat-Jacob H, Laguri C. 2015. C5-epimerase and 2-O-sulfotransferase associate in vitro to generate contiguous epimerized and 2-O-sulfated heparan sulfate domains. ACS Chem Biol. 10:1064–1071. 25594747 10.1021/cb501037a

[ref31] Sies H, Parnham MJ. 2020. Potential therapeutic use of ebselen for COVID-19 and other respiratory viral infections. Free Radic Biol Med. 156:107–112. 32598985 10.1016/j.freeradbiomed.2020.06.032 PMC7319625

[ref32] Stachtea XN, Tykesson E, van Kuppevelt TH, Feinstein R, Malmstrom A, Reijmers RM, Maccarana M. 2015. Dermatan sulfate-free mice display embryological defects and are neonatal lethal despite normal lymphoid and non-lymphoid organogenesis. PLoS One. 10:e0140279. 26488883 10.1371/journal.pone.0140279 PMC4619018

[ref33] Tucker AS, Slack JM. 2004. Independent induction and formation of the dorsal and ventral fins in *Xenopus laevis*. Dev Dyn. 230:461–467. 15188431 10.1002/dvdy.20071

[ref34] Tykesson E, Hassinen A, Zielinska K, Thelin MA, Frati G, Ellervik U, Westergren-Thorsson G, Malmstrom A, Kellokumpu S, Maccarana M. 2018. Dermatan sulfate epimerase 1 and dermatan 4-O-sulfotransferase 1 form complexes that generate long epimerized 4-O-sulfated blocks. J Biol Chem. 293:13725–13735. 29976758 10.1074/jbc.RA118.003875 PMC6120188

[ref35] Tykesson E, Mao Y, Maccarana M, Pu Y, Gao J, Lin C, Zaia J, Westergren-Thorsson G, Ellervik U, Malmstrom L, et al. 2016. Deciphering the mode of action of the processive polysaccharide modifying enzyme dermatan sulfate epimerase 1 by hydrogen-deuterium exchange mass spectrometry. Chem Sci. 7:1447–1456. 26900446 10.1039/c5sc03798k PMC4755500

[ref36] Wlad H, Maccarana M, Eriksson I, Kjellen L, Lindahl U. 1994. Biosynthesis of heparin. Different molecular forms of O-sulfotransferases. J Biol Chem. 269:24538–24541. 7929122

