# Supplementary material for: Inhibition of iduronic acid biosynthesis by ebselen reduces glycosaminoglycan accumulation in mucopolysaccharidosis type I fibroblasts
Source: Glycobiology. 2021 Jun 29;31(10):1319–29. doi: 10.1093/glycob/cwab066 (PMC8600295; doi:10.1093/glycob/cwab066)
Supplement: 20210608_Supplementary_data_resubmitted_cwab066 [file 20210608_supplementary_data_resubmitted_cwab066.docx]

**Supplementary Materials and Methods**

The *Dse*-MO (5’-GCT CCC CGA GTG TGA GTC CTC ATT G-3’) and standard control-MO (5’-CCT CTT ACC TCA GTT ACA ATT TAT A-3’) were obtained from Gene Tools LLC. To synthesize *nlacZ* mRNA, pCS2-*nlacZ* cDNA was linearized with NotI and transcribed with Sp6 RNA polymerase, using the mMessage Machine kit (Ambion). Unless otherwise stated, MOs were injected animally into all blastomeres at the two- or four-cell stage. A total of 45 ng MO was injected per embryo. For single injections, a quarter of the MO amount with 125 pg *nlacZ* mRNA as lineage tracer was used.

**Supplementary Figure legends**

Fig. S1. Dse/DS-epi1 knockdown reduces the development of neural crest-derived melanocytes and dorsal fin structures and suppresses neural crest cell migration in *Xenopus* embryos

(A and B) Microinjection of a morpholino oligonucleotide against Dermatan sulfate epimerase-1 (*Dse*-MO), but not a control-MO, induces small eyes, lack of dorsal fin structures (arrowheads) and less melanocytes (arrow).

(C and D) A single dorsal injection of *Dse*-MO, but not control-MO, inhibits the migration of *Twist*^+^ cranial neural crest cells (arrow) on the injected side in tailbud embryos. *nlacZ* mRNA was co-injected as lineage marker (red nuclei).

The proportion of embryos with the described phenotypes was as follows: A, 70/70; B, 71/114 (small head), 92/114 (reduced dorsal fin), 70/114 (less melanocytes); C, 47/47; D, 54/63. Each injection experiment was at least four times performed. br, branchial arch, ey, eye; hy, hyoid arch; ma, mandibular arch.

Fig. S2. Ebselen does not change GAG and CS/DS content in brain, kidney, and spleen

Five MPS-I mice were fed with food supplemented with ebselen and five MPS-I mice with control food for 10 weeks. At the end of the treatment period, GAGs were purified from the organs and quantified by the carbazole reaction (analytical triplicates). CS/DS was separately quantified by disaccharide fingerprint following chondroitinase ABC digestion (analytical duplicates).
