# Supplementary figures and images for: Inhibition of iduronic acid biosynthesis by ebselen reduces glycosaminoglycan accumulation in mucopolysaccharidosis type I fibroblasts
Source: Glycobiology. 2021 Jun 29;31(10):1319–29. doi: 10.1093/glycob/cwab066 (PMC8600295; doi:10.1093/glycob/cwab066)

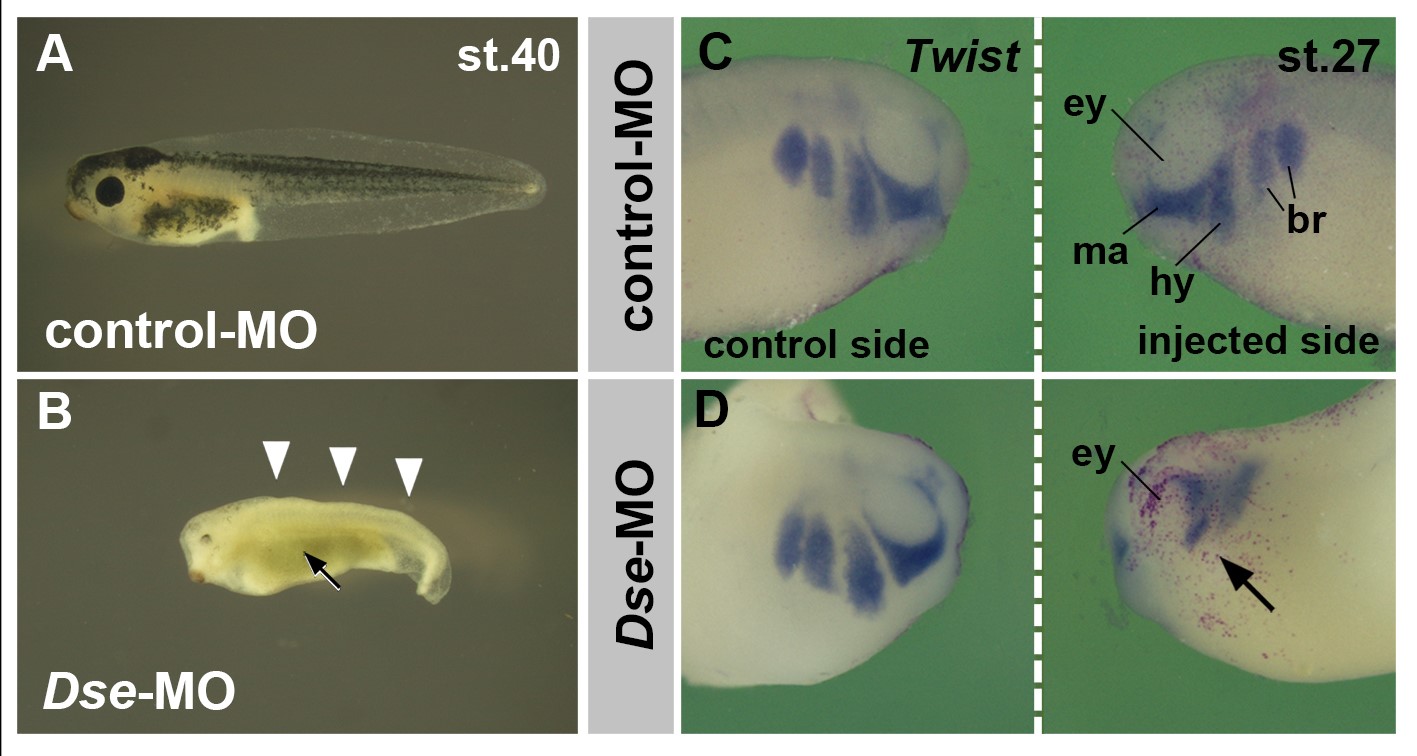

Supplement: Fig_S1_resubmitted_cwab066 [file fig_s1_resubmitted_cwab066.jpeg]

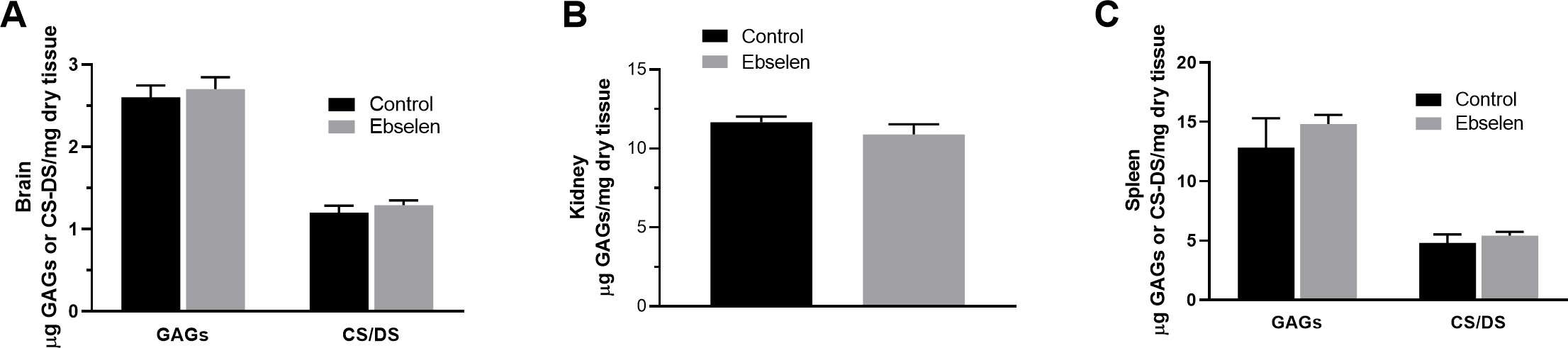

Supplement: Fig_S2_resubmitted_cwab066 [file fig_s2_resubmitted_cwab066.jpeg]
